# Prognostic role of albumin-bilirubin (ALBI) score and Child-Pugh classification in patients with advanced hepatocellular carcinoma under systemic treatment

**DOI:** 10.3332/ecancer.2024.1748

**Published:** 2024-08-27

**Authors:** Leonardo G da Fonsecaa, Marina Acevedo Zarzar de Melob, Thamires Haick Martins da Silveirac, Victor Junji Yamamotod, Pedro Henrique Shimiti Hashizumee, Jorge Sabbagaf

**Affiliations:** Department of Oncology, ICESP – Instituto do Cancer do Estado de Sao Paulo, University of Sao Paulo School of Medicine, Sao Paulo, SP 01246-000, Brazil; a https://orcid.org/0000-0002-0216-3618; b https://orcid.org/0000-0002-3031-7928; c https://orcid.org/0009-0000-8427-8592; d https://orcid.org/0000-0002-1422-0042; e https://orcid.org/0000-0002-9159-6756; f https://orcid.org/0000-0003-0715-4670

**Keywords:** carcinoma hepatocellular, liver neoplasms, liver function, liver cirrhosis, ascites, prognosis

## Abstract

Hepatocellular carcinoma (HCC) is a lethal malignancy associated with cirrhosis and liver dysfunction. The aim of this study is to characterize a cohort of patients with advanced HCC according to liver function-related variables and evaluate the prognostic significance of Child-Pugh (CP) and albumin-bilirubin (ALBI) scores. A database of 406 HCC patients treated between 2009 and 2023 was retrospectively evaluated. Clinical and laboratory parameters were collected to classify patients into ALBI and CP scores. Survival was estimated using the Kaplan-Meier method and multivariate models were used to evaluate prognosis prediction. In this cohort, 337 (83%) patients were classified as CP-A, while 69 (17%) as CP-B. Additionally, according to ALBI score, 159 (39.2%) individuals were categorised as ALBI-1, 233 (57.4%) as ALBI-2 and 14 (3.4%) as ALBI-3. A statistically significant association between both classifications was observed (*p* < 0.001). CP and ALBI scores were independently associated with prognosis (Hazard ratio = 2.93 and 1.66, respectively), with better survival for patients with CP-A (versus B) and ALBI-1 (versus -2 and -3). ALBI score showed better predictive performance versus CP (c Harrell´s C index = 0.65 versus 0.62; *p* = 0.008) and ALBI evolution during the first month of treatment was associated with overall survival. Additionally, ALBI score was able to define distinct prognostic subgroups within CP-A patients. In conclusion, liver function scores, such as ALBI and CP, have a clinically relevant prognostic role in patients with advanced HCC under systemic treatment. ALBI score is a more granular scoring scale than CP, and enables a more precise evaluation of patients with CP-A.

## Background

Hepatocellular carcinoma (HCC) is one of the leading causes of cancer-related deaths worldwide. HCC lethality is attributed to the high rate of late-stage diagnosis, and to the underlying liver cirrhosis, that is present in around 80% of patients [1].

Impaired liver function affects the prognosis of HCC patients across all stages. In individuals experiencing severe liver function impairment, the expected survival is less than 6 months, unless they qualify as potential candidates for liver transplantation [2, 3]. For patients in early or intermediate stages of HCC, the hepatic functional reserve limits surgical resection, ablation and intra-arterial therapies [4]. In advanced-stage HCC, cirrhosis compensation is a key factor in deciding the treatment strategy [3].

Systemic treatments, such as immunotherapy and targeted therapies, were approved for advanced HCC based on phase III trials that enrolled exclusively patients with Child-Pugh (CP) A [5–10]. This protocol restriction is justified by the competing risks of cirrhosis complications, altered drug metabolism and the increased risk of treatment-related adverse events, such as hepatotoxicity and bleeding. Therefore, the benefit of systemic treatment in patients with liver dysfunction is not clear, with scarce data showing a limited efficacy [11, 12].

Several models to quantify liver function are used in clinical trials and in daily practice. The CP score was originally proposed in the 1960s and further validated in different settings, including HCC prognostication [13–15]. CP score is a non-invasive indicator but relies on subjective measures such as grade of ascites and encephalopathy. Additionally, laboratory measurements of albumin, bilirubin and prothrombin time are graded in fixed cut-offs. The Albumin-Bilirubin (ALBI) score is another liver function index based solely on albumin and bilirubin levels, eliminating the need for subjective assessment of ascites and encephalopathy. The ALBI score was originally developed for HCC patients and validated in different stages, achieving accuracy in the detection of small changes in liver function [16–19].

The Barcelona Clinic Liver Cancer (BCLC) staging system is the most used guideline in the management of HCC. According to the BCLC algorithm, the decompensation indicators (ascites, encephalopathy, jaundice and variceal bleeding) define non-preserved liver function irrespective of CP classification [3]. The ALBI score can add additional granularity to the stratification of compensated patients [16–19].

The aim of this study is to characterize a cohort of patients with advanced HCC according to liver function-related variables, evaluate the concordance between CP and ALBI scores, and assess the prognostic significance of CP and ALBI scores in patients undergoing systemic treatment.

## Materials and methods

### Patients and methods

A database of HCC patients consecutively treated at Instituto do Cancer do Estado de Sao Paulo (Brazil) from July 2009 to June 2023 was retrospectively evaluated. The included population consisted of patients with HCC diagnosed on the basis of radiologic and/or histologic criteria [20] who initiated first-line systemic treatment according to local policy. All patients had an advanced (BCLC-C) or intermediate (BCLC-B) stage that was not amenable to locoregional treatment. The study was approved by the local Ethics Committee on 20th January 2020 and informed consent was waived due to the retrospective nature of the study (report 3.807.496).

Relevant data were collected from medical records including age, gender, performance status (PS) according to the Eastern Cooperative Oncology group (ECOG) scale, etiology, BCLC stage and serum laboratory parameters. Data were last updated on 12th-August 2023.

The ALBI grade was calculated using the following equation: linear predictor = (log_10_ bilirubin μmol/L × 0.66) + (albumin g/L × −0.085). The continuous linear predictor was further categorised into three different grades for prognostic stratification purposes: grade 1 (less than −2.60), grade 2 (between −2.60 and −1.39) and grade 3 (above −1.39) as previously described [19].

The CP score considers five variables: encephalopathy, ascites, serum bilirubin, albumin concentration and prothrombin time. Depending on the total points, CP is divided into class A (5–6 points), B (7–9 points) and C (10–15 points).

### Treatment and assessments

During the study period, sorafenib was the first-line treatment available to patients who were candidates for systemic treatment at the institution. Sorafenib was administered orally at an initial dose of 400 mg twice daily, which could be modified upon the development of adverse events according to type and severity. Clinical and laboratory assessments were performed at baseline and monthly, and radiologic evaluation was done bimonthly. Treatment was continued until symptomatic progression, radiological progression, treatment intolerance or death.

### Statistical analysis

Quantitative variables were expressed as median and interquartile range (IQR 25th–75th percentiles). Categorical variables were described as absolute frequencies and percentages (%). Fisher’s exact test was used to compare categorical variables. Time-to-event variables were described using the Kaplan-Meier method with median and their 95% confidence interval (CI). Survival functions were compared using the log-rank test.

Hazard ratios (HR) and 95%CI were estimated using a Cox regression model adjusted for the BCLC stage (because of the well-established prognostic role of this staging system) and ascites (because the ALBI score does not include ascites). We performed a landmark analysis at 1 month of treatment, in which we classified the cohort into groups according to the evolution of ALBI grade into: ‘stable’ (same grade at baseline and 1 month), ‘decline’ (worse grade at 1 month than at baseline) and ‘improvement’ (better grade at 1 month than at baseline).

The prognostic accuracy of the CP and ALBI models was evaluated using Harrell´s C concordance index, in which a higher C index indicates greater accuracy and agreement between predicted and expected outcomes [21]. All tests were two-sided with a significant level of 0.05. Analyses were performed using Stata software version 15.1 (StataCorp, College Station, TX).

## Results

### Baseline characteristics

A total of 406 patients were included. The median age was 62.9 years (IQR; 55.5–69.5), most patients were male (*n* = 304; 74.9%), ECOG-PS 0 (*n* = 209; 51.5%) and BCLC-C stage (*n* = 292; 72.9%). Two hundred and eighteen (53.7%) patients received prior locoregional treatments for HCC. Baseline demographic and clinical characteristics are summarised in Table 1.

### Liver function assessments

At baseline, 337 (83%) patients were classified as CP-A and most were CP A5 (*n* = 241, 59.4%). Only 69 (17%) patients were classified as CP-B. When classified according to the ALBI score, 159 (39.2%) were ALBI-1, 233 (57.4%) were ALBI-2 and 14 (3.4%) were ALBI-3 (Table 2).

Among the patients classified as CP-A, 158 (46.9%) were ALBI-1 and 175 (51.9%) were ALBI-2. Among patients classified as CP B, 58 (84.1%) patients were ALBI-2, 10 (14.4%) patients were ALBI-3 and only 1 patient was ALBI-1. There was a significant association between CP and ALBI classifications (Pearson chi-square = 69.9; *p* < 0.001) (Table 2). Finally, 41 (10.1%) patients presented ascites detected clinically and/or radiologically. Similarly, there was a significant association between the ALBI score and the presence of ascites (Pearson chi-square = 20.1; *p* < 0.001).

### Liver function scores and prognosis

At the last follow-up update, 359 (88.4%) patients had died, 11 (2.7%) were lost to follow-up and 36 (8.9%) were alive. The median follow-up was 9.1 months (IQR; 4.1–19.0). The median overall survival (OS) of the entire cohort was 9.4 months (95%CI: 8.5–10.6 months).

**Figure 1.**
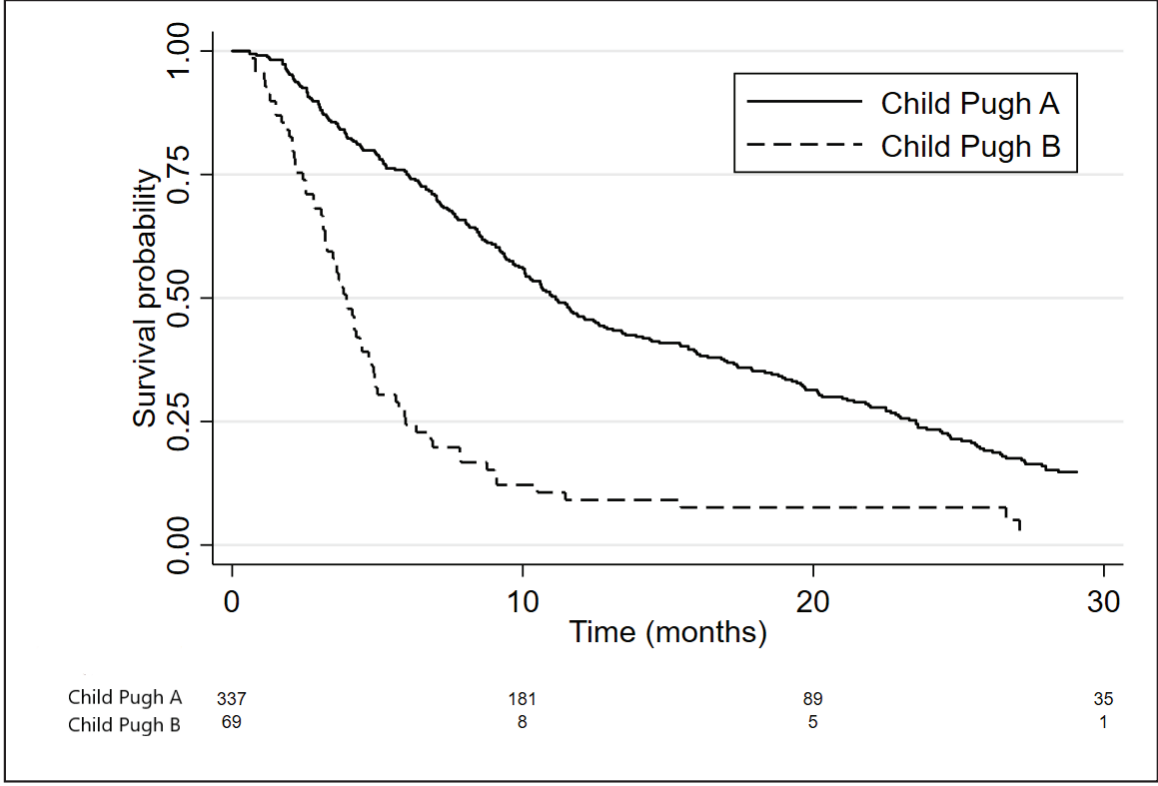
Kaplan Meier curves showing the survival of patients according to CP score.

CP-A and CP-B patients presented a median OS of 11.1 months (95%CI 10.1–12.6) and 3.9 months (95%CI 3.2–4.7), respectively, with an adjusted HR = 2.93; 95%CI: 2.2–3.9; *p* < 0.001 (Figure 1).

**Figure 2.**
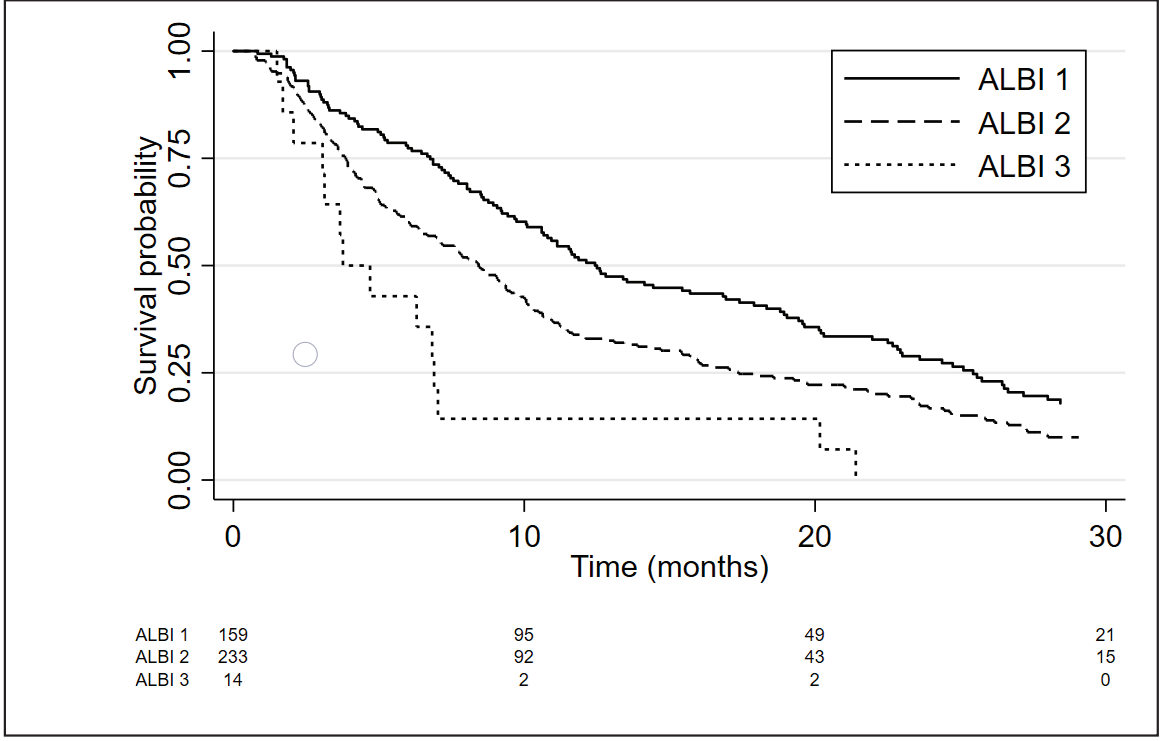
Kaplan Meier curves showing the survival of patients according to ALBI score.

The median OS of ALBI-1, ALBI-2 and ALBI-3 groups was 12.5 months (95%CI 10.6–16.9), 8.4 months (95%CI 6.97–9.67) and 3.8 months (95%CI 2.1–6.9), respectively, with a HR = 1.66; 95%CI 1.4–2.0; *p* < 0.001 (Figure 2). ALBI score showed a better prediction performance versus CP in the present cohort (c Harrell´s C 0.65 versus 0.62; *p* = 0.008).

Patients with clinical and/or radiological ascites at baseline (*n* = 41) presented an inferior median OS of 3.5 months (95%CI 2.4–4.2) versus 10.6 months (95%CI: 9.4–11.7). Ascites was also an independent prognostic factor when adjusting for both ALBI (HR = 1.42, 95%CI: 1.12–1.69; *p* < 0.001) and CP score (2.41; 95%CI: 1.56–3.12; *p* < 0.001).

**Figure 3.**
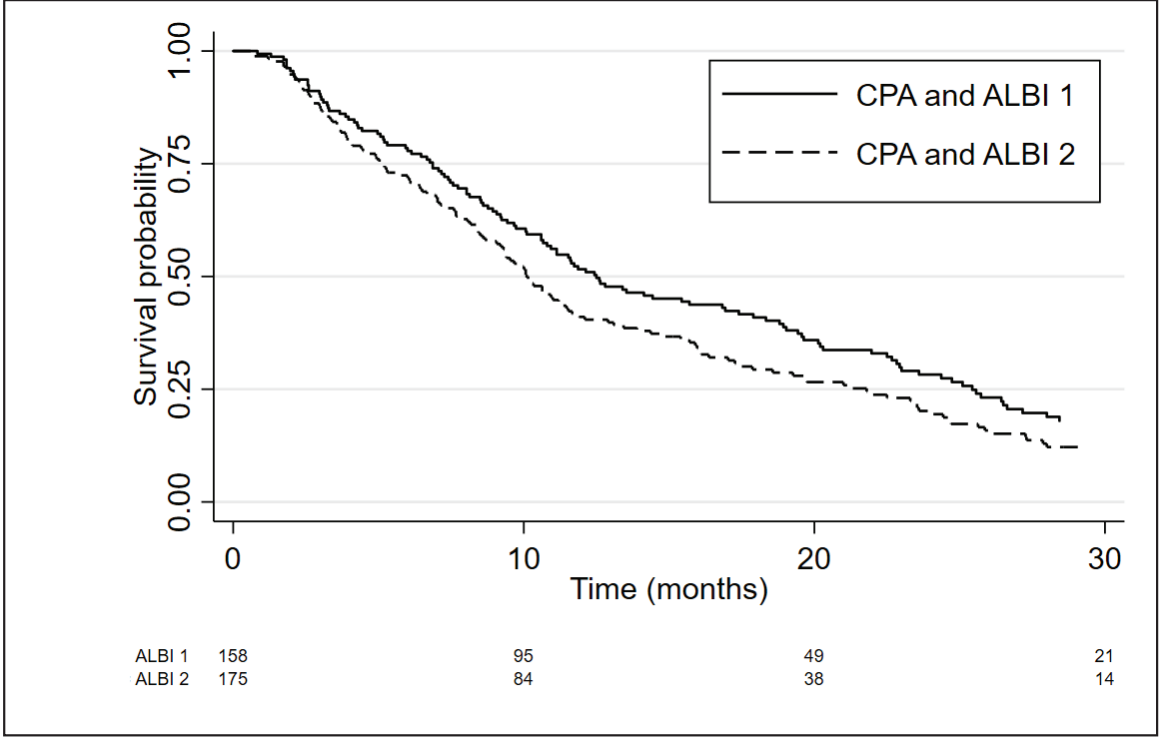
Kaplan Meier curves of CPA patients according to ALBI score.

Considering only patients with CP-A, the ALBI score was able to differentiate two prognostic subgroups. Patients with CP-A and ALBI-1 (*n* = 158) presented a median OS of 12.5 months (95%CI 10.6–16.9) and patients with CP-A and ALBI-2 (*n* = 175) had a median OS of 10.1 months (95%CI 8.7–11.5), with an adjusted HR = 1.36; 95%CI: 1.07–1.72; *p* = 0.012 (Figure 3 and Table 3).

**Figure 4.**
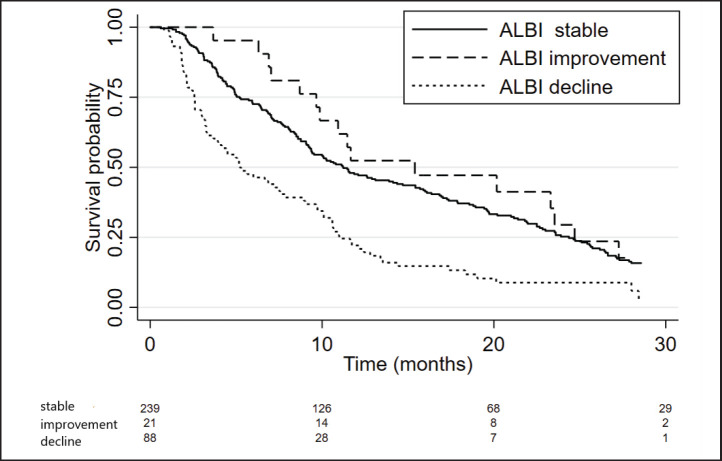
Kaplan Meier curves of patients according to the ALBI score evolution within the first month of treatment (stable versus improvement versus decline).

**Figure 5.**
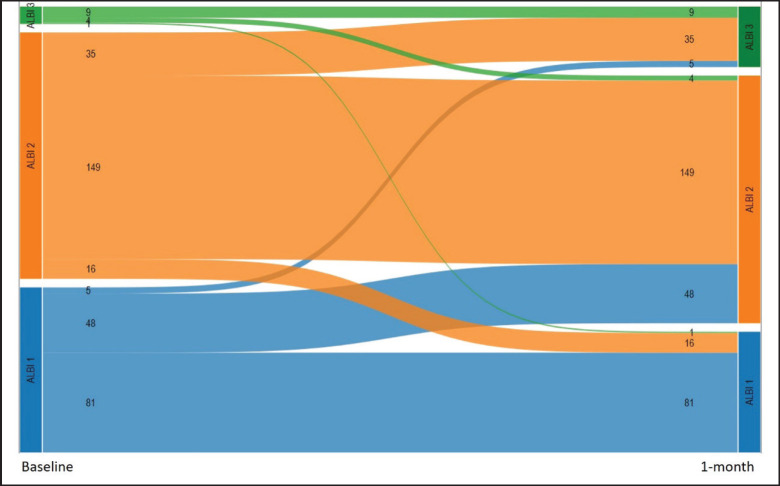
Alluvial graph showing the number patients with each ALBI grade at baseline (left) and after 1 month (right), and how the ALBI grade evolved during the first month (58 patients with missing ALBI score at 1 month are not represented).

We also performed a landmark analysis at 1 month to describe the prognosis of patients according to the evolution of the ALBI score during the first month of systemic treatment. Fifty-eight patients were not included in this analysis due to missing albumin and/or bilirubin measurement at 1 month. Patients who improved the ALBI score at 1 month (*n* = 21) had a similar OS compared to patients who maintained a stable ALBI score (*n* = 239) (15.4 versus 11.2 months; *p* = 0.611), while patients who presented worsened ALBI score at 1 month (*n* = 88) had significantly inferior OS (5.2 months, 95%CI: 3.7–7.9; *p* < 0.001 HR 2.1, 95%CI: 1.6–2.8) (Figures 4 and 5).

## Discussion

In this cohort of patients with HCC undergoing systemic treatment, liver function was a key prognostic factor. We validated the well-known role of baseline CP classification and highlighted that the ALBI score can be a more accurate model to stratify liver function in patients with advanced HCC. In addition, the evolution of the ALBI score during the treatment could refine the prognostic assessment, particularly within the first month of treatment.

CP score is traditionally adopted in clinical trials testing systemic therapies in advanced HCC. Consequently, it is widely used in clinical routines to guide practical decisions [1, 22]. However, CP classification has some drawbacks. Some variables included in the CP classification can be subjective, such as hepatic encephalopathy and ascites. Additionally, all variables are given the same weight and cut-offs are empirical. For example, the presence of ascites alone is recognised as a sign of decompensated cirrhosis, while a decline in serum albumin is not an independent indicator of decompensation that affects the prognosis in a similar weight [23]. This is the reason we analysed the presence of ascites separately and adjusted the regression models with this variable.

ALBI score was designed by Johnson *et al* [19] from a cohort of 1,313 HCC patients of all stages from Japan, and the model was validated in the original study in around 5,000 patients across different stages and geographic regions. ALBI showed a favourable performance comparing to CP, mainly in patients with CP-A [19]. Since then, several studies have proven that ALBI can be a reliable tool to assess liver function [18]. ALBI score was mostly validated in patients that received treatment interventions, so it is reasonable to assume that this model performs better in patients without acute or severe liver decompensation. However, clinical conditions other than liver deterioration may limit the predictive ability of the ALBI score, such as biliary obstruction, hemolysis, malnutrition and excessive albumin loss. Therefore, a thorough clinical assessment is required to interpret and apply the ALBI score. In our cohort, all patients received systemic treatment according to the local policy. Patients with severe decompensation, such as tense ascites, recent variceal bleeding, active infection or clinical deterioration are routinely referred to supportive care instead of receiving active treatment. Therefore, most of our cohort presented ECOG PS 0-1, CP-A, no ascites and were classified as ALBI-1 or -2.

Both CP and the presence of ascites had a significant association with the ALBI score, showing that there is a good concordance across different models to assess liver function. We updated our previous observation that CP-A patients had better survival compared to CP-B as expected [12], while ALBI-1 had better survival compared to ALBI-2 and ALBI-3. ALBI 3 showed poorer prognosis with a median OS of less than 4 months, highlighting that this subgroup might not have benefited from any treatment due to low hepatic reserve and should be considered to palliative supportive care.

Patients with CP-A are globally considered the optimal candidates to systemic treatment and this is an inclusion criterion in all large clinical trials [7–9]. However, in our cohort, patients with CP-A could be further subdivided in prognostic subgroups according to ALBI score. CP-A and ALBI-1 had better prognosis comparing to CPA and ALBI-2. Our finding suggests that ALBI should be adopted to refine treatment allocation and survival prediction both in clinical trials and in daily practice. These observations validate the original description of ALBI by Jonhson *et al* [19].

A relevant finding of our study is that the evolution of ALBI score within the first month of systemic treatment can further refine the prognostic evaluation. This result suggests that alterations in albumin and/or bilirubin during treatment may reflect alterations in liver function and finally impact prognostic. Patients who improved their ALBI score in the first month had similar survival to patients who started treatment with ALBI-1. Kuzuya *et al* [24] also showed that the evolution of ALBI score during treatment with cabozantinib impacts survival outcomes. This indicates the need for individualised follow-up strategies in patients at risk for liver decompensation and the adoption of measures to improve liver function, such as antiviral treatment, alcohol abstinence, avoidance of hepatotoxic drugs and encephalopathy prophylaxis.

A limitation of our study is the retrospective nature, which prevents a precise identification of confounding factors and detailed information of additional tests that are not routinely performed in advanced HCC patients (such as elastography and endoscopy). Furthermore, most patients started treatment in a period when sorafenib was the standard first-line treatment before the approval of immunotherapy combinations.

## Conclusion

In conclusion, we showed that liver function models, such as ALBI and CP scores, have a clinically relevant prognostic role in advanced HCC under systemic treatment. ALBI score is a more granular scoring scale and enables a more precise evaluation of patients with CP-A. The early changes of ALBI score during HCC systemic treatment identify different outcomes. This finding indicates that the oncologic outcome is closely related to the hepatic reserve in advanced HCC.

## List of abbreviations

ALBI, Albumin-bilirubin; BCLC, Barcelona clinic liver cancer; CI, Confidence interval; CP, Child-Pugh; ECOG, Eastern Cooperative Oncology group; HCC, Hepatocellular carcinoma; HR, Hazard ratio; IQR, Interquartile range; NR, Not-reached; OS, Overall survival; PS, performance status.

## Conflicts of interest

Authors declare that they have no competing interests.

## Funding

This research did not receive any specific grant from funding agencies in the public, commercial or not-for-profit sectors.

## Availability of data and materials

The dataset collected and analysed in the present study are available from the corresponding author on request.

## Author contributions

LGF, MZ and TH: conceptualization. LGF, MZ, TH and VJ: data curation and formal analysis. LGF, MZ and TH: methodology. LGF, MZ, TH and VJ: investigation and methodology. LGF, MZ, TH and VJ: writing the manuscript. LGF, MZ, TH, VJ, PH review & editing. All authors have read and approved the manuscript.

## Ethics approval and consent to participate

The procedures followed were in accordance with the ethical standards of the responsible committee on human experimentation (institutional and national) and with the Helsinki Declaration of 1964 and later versions.

The Research Ethics Committee of Hospital das Clinicas da *Faculdade de Medicina da Universidade de São Paulo* (HCFMUSP) approved the study protocol (protocol report identification: 3.807.496; 20th January 2023).

## Patient consent for publication

Patient consent for publication was waived by the local Research Ethics Committee (Comite de Etica para Analise de Projetos de Pesquisa CAPPesq- Hospital das Clinicas da *Faculdade de Medicina da Universidade de São Paulo* – HCFMUSP).

## Figures and Tables

**Table 1.** Baseline and demographic characteristics of the cohort.

Baseline characteristics	*N* = 406
Median age, (IQR)	62.9 years (IQR; 55.5–69.5)
Gender	
• Male, *n* (%)	304 (74.9%)
• Female, *n* (%)	102 (25.1%)
Chronic liver disease etiology	
• No hepatopathy, *n* (%)	10 (2.5%)
• Hepatitis C, *n* (%)	162 (39.9%)
• Hepatitis B, *n* (%)	52 (12.1%)
• Alcohol, *n* (%)	61 (15.0%)
• MASLD, *n* (%)	42 (10.3%)
• Others	79 (19.4%)
Previous treatment^*^	
• Transplant, *n* (%)	23 (5.7%)
• Resection, *n* (%)	51 (12.6%)
• Ablation, *n* (%)	40 (9.9%)
• TACE, *n* (%)	179 (44.1%)
• No previous treatment, *n* (%)	188 (46.3%)
BCLC stage	
• B, *n* (%)	110 (27.1%)
• C, *n* (%)	296 (72.9%)
Macrovascular invasion, *n* (%)	167 (41.1%)
Extrahepatic spread, *n* (%)	166 (40.9%)
ECOG PS, *n* (%)	
• 0–1, *n* (%)	363 (89,2%)
• 2, *n* (%)	44 (10.8%)
Alpha-fetoprotein, median (IQR)	193 (17–4.3607)

* Some patients received more than 1 previous treatment. IQR: interquartile range; ECOG: Eastern cooperative oncology group; MASLD: metabolic associated steatotic liver disease; TACE: transarterial chemoembolization; BCLC: Barcelona clinic liver cancer

**Table 2.** Subgroups according to liver function scores ALBI and CP.

	ALBI 1	ALBI 2	ALBI 3	Total
CP A	158 (99.4%)	175 (75.1%)	4 (28.6%)	337 (83%)
CP B	1 (0.6%)	58 (24.9%)	10 (71.4%)	69 (17%)
Total	159 (100%)	233 (100%)	14 (100%)	406 (100%)

Pearson *χ*^2^ = 69.8978; *p* < 0.001

**Table 3.** Outcomes of subgroups based on CP and ALBI cores.

	*N*	Median survival (95%CI)	Adjusted HR (95%CI); *p* value
CP subgroups			
CP A	337	11.1 months (10.1–12.6)	1 (reference)
CP A5	241	13.4 monhs (11.5–17.4)	-
CP A6	96	7.0 months (5.3–8.6)	-
CP B		3.9 months (3.2–4.7)	2.93 (2.22–3.89); *p* < 0.001
CP B7	45	3.9 months (3.3–4.9)	-
CP B8	18	3.2 months (2.1–4.4)	-
CP B9	6	1.9 months (1.1–NR)	-
			
ALBI subgroups			
ALBI 1	159	12.5 months (10.6–16.9)	1 (reference)
ALBI 2	233	8.4 months (6.9–9.7)	1.58 (1.26–1.97) *p* < 0.001
ALBI 3	14	3.8 months (2.1–6.9)	3.40 (1.9–6.1), *p* < 0.001
			
ALBI subgroups in CP-A			
ALBI 1 and CP-A	158	12.5 months (10.6–16.9)	1 (reference)
ALBI 2 and CP-A	175	10.1 months (8.7–11.5)	1.35 (1.07–1.72); *p* = 0.012
ALBI 3 and CP-A	4	7.0 months (6.9–NR)	*

* Not analysed due to small sample size. CP: Child_Pugh; HR: Hazard ratio; CI: Confidence interval; NR: not reached
